# Ischaemia-reperfusion injury in photodynamic therapy-treated mouse tumours

**DOI:** 10.1038/sj.bjc.6600792

**Published:** 2003-03-04

**Authors:** M Korbelik, J Sun, H Zeng

**Affiliations:** 1 British Columbia Cancer Agency, Vancouver, BC, Canada

**Keywords:** photodynamic therapy, ischaemia-reperfusion injury, xanthine oxidase, visible reflectance spectroscopy, mouse fibrosarcoma

## Abstract

Prompted by the observation of ischaemia development during the treatment of tumours by photodynamic therapy (PDT) that is typically followed by a restoration of tumour blood flow and by the indications of secondary superoxide generation after PDT, we aimed in this study to obtain evidence of the induction of ischaemia-reperfusion (I/R) injury in PDT-treated tumours. Using subcutaneous mouse FsaR fibrosarcoma model and Photofrin-based PDT treatment, we have examined the activity of xanthine oxidase (XO, a key enzyme in the I/R injury development) in tumours before and after the therapy. Compared to the levels in nontreated tumours, there was a five-fold increase in the activity of this enzyme in tumours excised immediately after PDT. This burst of elevated XO activity declined rapidly, returning to the pretreatment levels within the next 30 min. Visible reflectance spectroscopy confirmed the occurrence of a PDT-induced strong but temporary reduction in tumour oxygenation. The administration of XO inhibitor oxypurinol prevented this PDT-induced rise in XO activity. The oxypurinol treatment also decreased the extent of neutrophil accumulation in PDT-treated tumours and reduced the level of PDT-mediated cures. These results demonstrate the induction of I/R injury in PDT-treated tumours, and show that it can contribute to the therapy outcome. Since I/R injury is a well-recognised proinflammatory insult, we suggest that its induction in PDT-treated tumours promotes the development of inflammatory response that has become established as a key element of the antitumour effect of PDT.

Recent insights into the mechanisms of photodynamic therapy (PDT)-mediated destruction of solid tumours have emphasised the important role played by the induced inflammatory response for the outcome of this therapy (Dougherty *et al*, 1998; Korbelik and Cecic, 2003; Sun *et al*, 2002). Evidence has accumulated documenting various inflammation-specific events following the treatment of tumours with PDT, including: (i) proinflammatory changes in vascular endothelium, (ii) complement activation and engagement of other plasma cascade systems (kinin-generation, coagulation, and fibrinolysis), (iii) release of inflammatory cytokines and chemokines, arachidonic acid metabolites and various other inflammatory mediators, (iv) activation of poly(ADP-ribose)polymerase and NF*κ*B upregulation, and (v) invasion of inflammatory cells (Ryter and Gomer, 1993; Cecic and Korbelik, 2002; Korbelik and Cecic, 2003; Sun *et al*, 2002). The transcription factor NF*κ*B is now a recognised key regulator of inflammatory response (Lentsch and Ward, 2000).

Ischaemia-reperfusion (I/R) injury is known as a potent instigator of inflammatory response responsible for severe tissue damage in a variety of common pathological conditions, including stroke, myocardial infarction, pulmonary and haemorrhagic shock, acute kidney and liver failure, and organ transplant rejection (Hernandez *et al*, 1987; Zimmerman and Granger, 1994; De Greef *et al*, 1998). Tissue ischaemia is associated with the conversion of xanthine dehydrogenase into oxidant-producing xanthine oxidase (XO), while concomitantly hypoxanthine accumulates because of the breakdown of ATP (Parks *et al*, 1988; Zimmerman and Granger, 1994). At the time of reperfusion, sudden reintroduction of oxygen enables XO to induce the formation of xanthine from hypoxanthine, which is accompanied with an intense release of reactive oxygen species, primarily superoxide anion (Parkins *et al*, 1998). The induced oxidative stress at the level of vascular endothelium promotes complement activation and elicits a series of inflammatory events culminating in a massive invasion of activated neutrophils and other inflammatory cells into the previously ischaemic area (Hernandez *et al*, 1987; De Greef *et al*, 1998; Kilgore *et al*, 1999).

In an earlier work (Korbelik *et al*, 2000), we found indications that I/R injury may play a role in the response of tumours to PDT. A typical pattern of blood flow alterations in PDT-treated tumours consists of an initial marked drop that tends to recover after photodynamic light treatment, and such conditions are conducive to the induction of I/R injury. When examining the possibility of superoxide generation during the reperfusion episode, we found that the administration of superoxide dismutase (SOD) immediately after PDT resulted in a decrease in tumour cure rates (Korbelik *et al*, 2000). Since I/R injury, if indeed inflicted in PDT-treated tumours, would be of a considerable relevance for the development of inflammatory responses, microvascular dysfunction and tumour cures, our objective in the present study was to obtain more conclusive evidence that would support the induction of this insult. We demonstrate that PDT results in a marked elevation in the activity of XO (a key enzyme that hallmarks the I/R process) in treated tumours, and show that XO inhibition attenuates the neutrophil infiltration into PDT-treated tumours and decreases tumour cure rates.

## MATERIALS AND METHODS

### Tumour model

Subcutaneous FsaR fibrosarcomas (Volpe *et al*, 1985) were inoculated by implanting 1 × 10^6^ *in vitro* expanded tumour cells into the lower sacral region of syngeneic C3H/HeN mice. Tumours were used for experiments when reaching 6–8 mm in largest diameter with thickness around 2 mm. All animal procedures were conducted according to the approval issued by The Animal Ethics Committee of the University of British Columbia and meet the standards required by the UKCCR guidelines (Workman *et al*, 1998).

### PDT protocol

Photofrin (porfimer sodium, Axcan Pharma Inc., Mont-Saint-Hilaire, Quebec, Canada) was administered intravenously at 10 mg kg^−1^ at 24 h prior to the delivery of light generated by a high throughoutput fibre illuminator (Sciencetech Inc., London, Ontario, Canada) equipped with a 150 Q QTH lamp with integrated ellipsoidal reflector and 630±10 nm interference filter. The light was delivered through an 8-mm core diameter liquid light guide model 77638 (Oriel Instruments, Stratford, CT, USA). The power density achieved for monodirectional superficial illumination of tumours and ∼1 mm of surrounding normal tissue was around 110 mW cm^−2^. During PDT light treatment, the mice were held unanaesthetised in restraining holders. For the evaluation of tumour cure or regrowth, the mice (eight per treatment group) were, after PDT, examined every second day for signs of tumour growth. Tumour cure was defined as no sign of recurrence at 90 days post-PDT. The XO inhibitor oxypurinol, purchased from Sigma Chemical Co. (St Louis, MO, USA), was dissolved in phosphate-buffered saline and administered intraperitoneally at 17 mg/kg^−1^. The ethical guidelines were followed that meet the above-mentioned standards (Workman *et al*, 1998).

### Measurement of XO activity

Amplex™ Red Xanthine/Xantine Oxidase Assay Kit (Molecular Probes, Eugene, OR, USA) was used for the measurement of XO activity in homogenates of the excised FsaR tumours. Briefly, this assay is based on the activity of hydrogen peroxide (formed by spontaneous degradation of superoxide, which is a major product in XO-mediated oxidation of hypoxanthine) that in the presence of horseradish peroxidase reacts stoichiometrically with Amplex Red reagent to generate the red-fluorescent oxidation product, resorufin. Resorufin fluorescence was measured in a fluorescence microplate reader using 530 and 590 nm wavelengths for excitation and detection, respectively. The results expressed in mU h^−1^ mg^−1^ of tumour tissue were derived by preparing a XO standard curve. In obtaining the excised tumours, we followed the ethical guidelines that meet the above-mentioned standards (Workman *et al*, 1998).

### Reflectance spectroscopy

The reflectance spectra were measured with a fibre optic spectrometer system developed in our laboratory (Zeng *et al*, 1995). A tungsten lamp is used for illumination through one branch of a bifurcated fibre bundle. Another branch of the fibre bundle collects and transmits the reflected light from the tissue to a spectrometer (Ocean Optics, FL, USA, model USB 2000) for spectral analysis. The fibre bundle has a holder to position itself at 45° to the skin surface to avoid the specular (mirror) reflection so that only the diffuse reflected light, which has gone into the tissue and sampled the tumour, was collected. The data acquisition of each spectrum is completed in less than 1 s and the measurements generate no significant PDT effect to the tissue. The spectral signals between 500 and 600 nm were used to assess the blood oxygenation status of the probed tissue volume. The ethical guidelines were followed that meet the above-mentioned standards (Workman *et al*, 1998).

### Flow cytometry

Tumour neutrophil levels were assessed using a flow cytometry protocol described in detail elsewhere (Cecic *et al*, 2001). Briefly, the excised tumours were dissociated into single-cell suspensions and the cells were stained with fluorescein isothiocyanate (FITC)- or phycoerythrin (PE)-conjugated monoclonal antibodies raised against specific murine membrane markers. Neutrophils were identified as cells stained positively for myeloid differentiation antigen GR1 (Ly-6G) and negatively for macrophage-specific antigen F4/80. Flow cytometry was performed with a Coulter Epics Elite ESP (Coulter Electronics, Hialeah, FL, USA) using standard techniques. In obtaining the excised tumours, we followed the ethical guidelines that meet the above-mentioned standards (Workman *et al*, 1998).

### Statistical analysis

The unpaired Student's *t*-test was applied to test the difference between means for the data from XO measurement and flow cytometry. Log-rank test was used for the tumour response evaluation. The difference with *P*<0.05 was considered statistically significant.

## RESULTS

**Figure 1:**
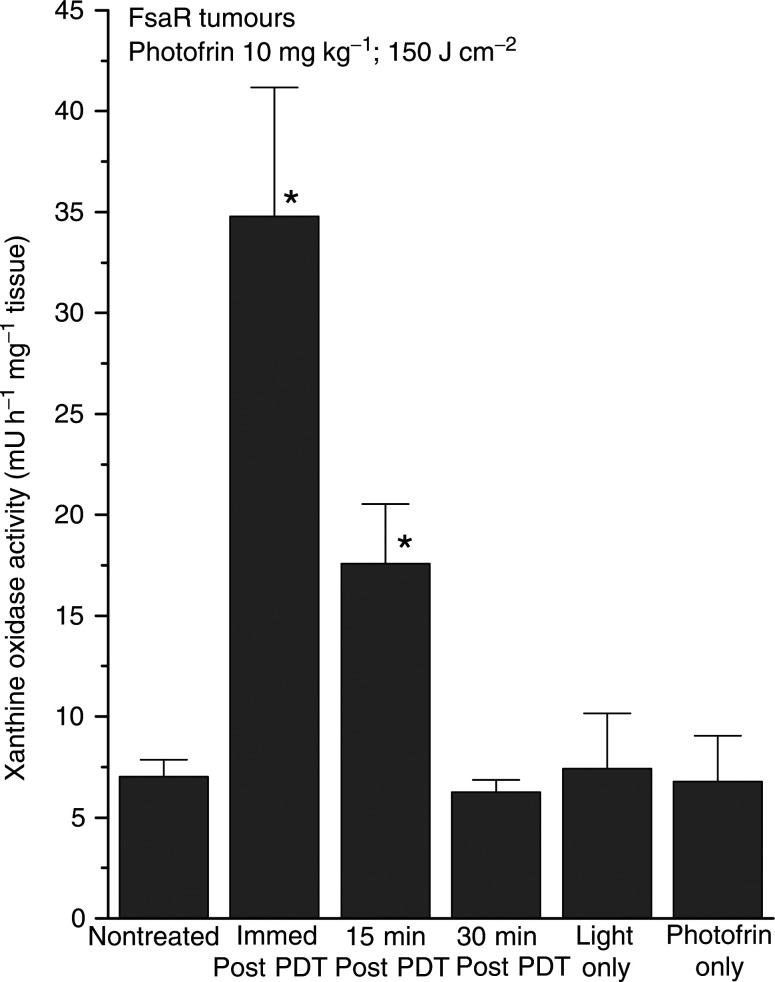
The effect of PDT on XO activity in FsaR tumours. Subcutaneous FsaR tumours growing in C3H/HeN mice were treated by PDT (Photofrin 10 mg kg^−1^ followed 24 h later by 150 J cm^−2^). The tumours were excised either immediately post-PDT light treatment, or 15 or 30 min later. Their homogenates were used for the determination of XO activity as described in Materials and Methods. The samples were also prepared from nontreated tumours, tumours from mice not given Photofrin excised immediately after light treatment (light only), and those from mice given Photofrin 24 h earlier but not treated with light (Photofrin only). Bars represent s.d., *n*=4; ^*^depicts statistically significant difference from the level in nontreated tumours (*P*<0.01).

The activity of XO determined in the homogenates of nontreated FsaR tumuors was around 7 mU h^−1^ mg^−1^ of tumour tissue (Figure 1

). No significant change in this level was detected in the light-only and Photofrin-only treatment control groups. However, a five-fold increase in XO activity was found in the tumours exposed to Photofrin-based PDT and excised immediately after the termination of light delivery. The activity of this enzyme was still elevated, but at a lower level, in tumours excised at 15 min post-PDT, while it dropped to the control levels in tumours excised at 30 min post-PDT.

**Figure 2:**
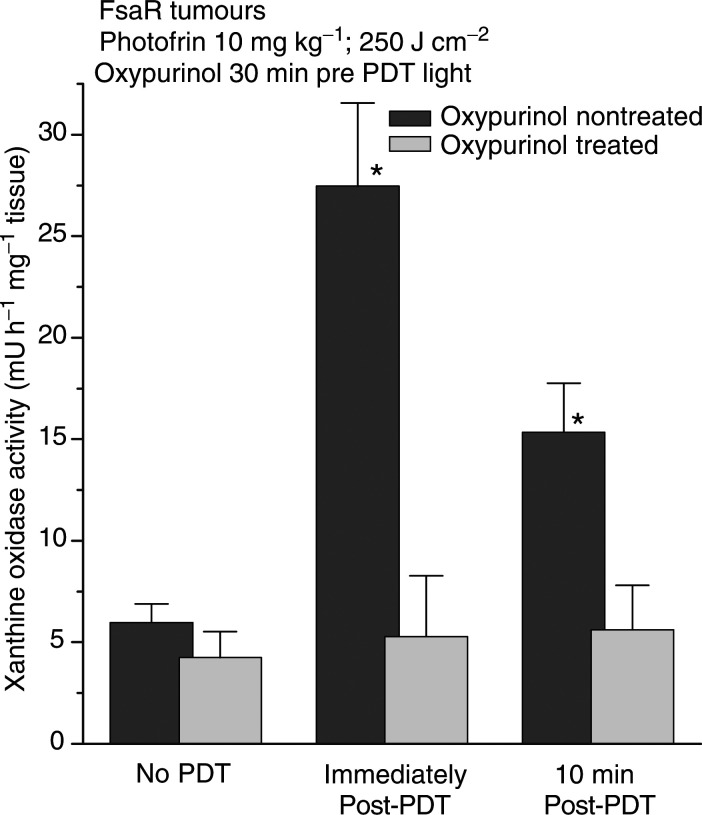
The effect of oxypurinol pretreatment on XO activity in PDT-treated FsaR tumours. Subcutaneous FsaR tumours were treated with PDT and the samples prepared for XO measurement as described in Figure 1, except that the time of excision was either immediately after PDT light treatment or 10 min later. Oxypurinol (17 mg kg^−1^) was injected intraperitoneally 30 min before the onset of light treatment. Bars are s.d., *n*=4; ^*^depicts statistically significant difference from the level in nontreated tumours (*P*<0.01).

In additional experiments, we examined the effect of oxypurinol, a specific inhibitor of XO (Granger *et al*, 1986; Spector *et al*, 1986). Although the light dose was increased from a moderately curative 150 J cm^−2^ (when used with 10 mg kg^−1^ of Photofrin) to a highly curative 250 J cm^−2^, there was no further increase in the XO activity in tumours excised immediately post-PDT (the average level was in fact somewhat lower) (Figure 2

). A group of tumours was also excised at 10 min post-PDT and the results show that the XO activity markedly declined in this short time period. The administration of oxypurinol at 30 min before PDT light treatment completely prevented the PDT-induced burst in XO activity, while oxypurinol has not significantly affected the activity of this enzyme in nontreated tumours.

**Figure 3:**
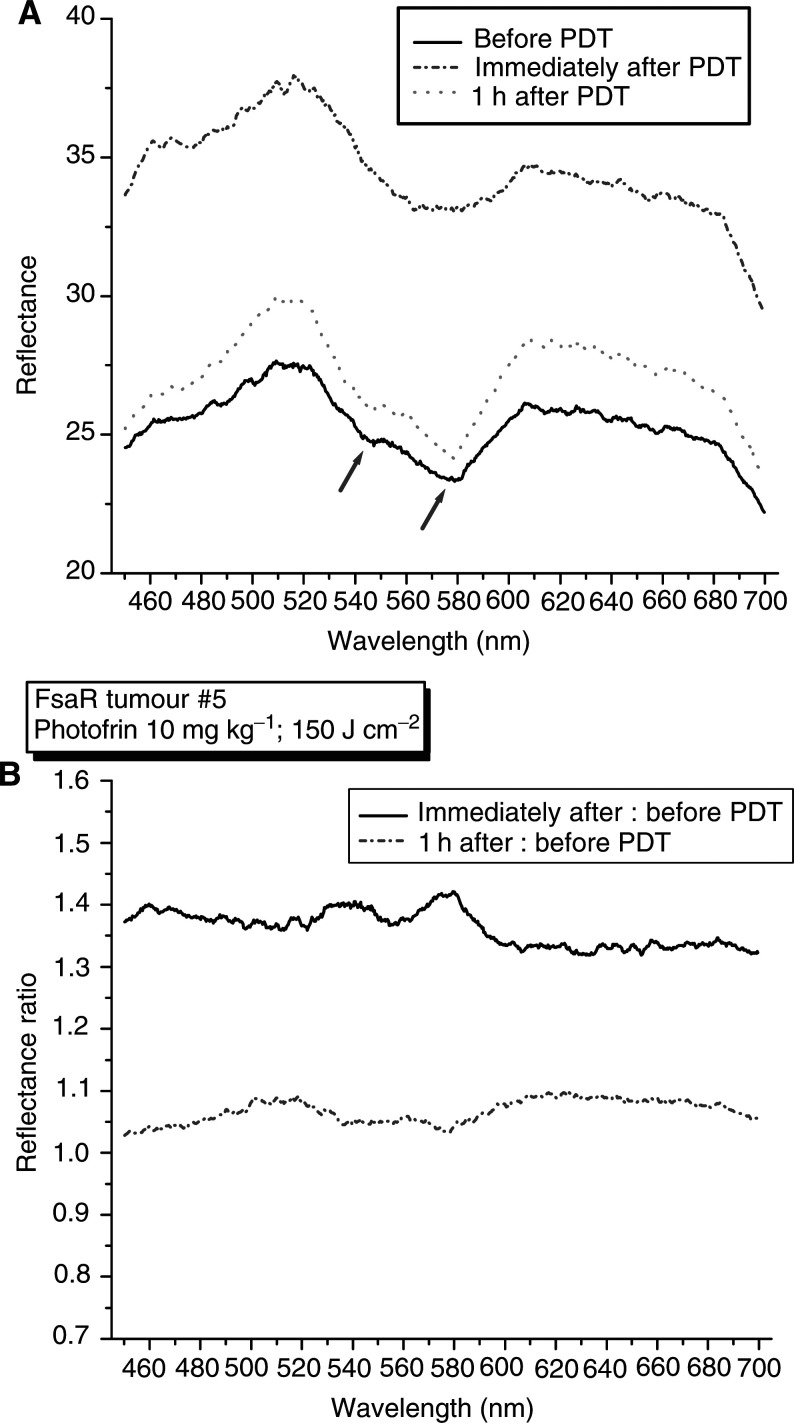
PDT-induced changes in the oxygenation of FsaR tumors monitored by reflectance spectroscopy. Subcutaneous FsaR tumours (six in total) were treated by Photofrin-based PDT as described for Figure 1. Visible light reflectance spectroscopy was performed with each tumour immediately before and immediately after PDT light delivery, as well as at 1 h after the termination of PDT light treatment. The results obtained with a representative tumour are shown as (**A**) reflectance spectra or (**B**) reflectance spectra ratios. The arrows identify characteristic troughs at 542 and 577 nm produced by oxyhaemoglobin.

An obvious cause for the observed changes in XO activity would be a temporary decline in the oxygenation of PDT-treated tumours. To verify this, we monitored the oxygenation status in a group of six subcutaneous FsaR tumours starting before PDT and extending to 1 h after PDT using a noninvasive visible reflectance measurement. Very similar results were obtained with all the tumours and a representative example is shown in Figure 3

. The reflectance spectrum recorded with the tumour before PDT has the characteristic troughs (dips) at 542 and 577 nm (arrows) that result from the secondary absorption bands of oxyhaemoglobin (Figure 3A). The spectrum obtained with the same tumour immediately after PDT exhibits a striking absence of the 542 and 577 nm troughs, which shows that oxyhaemoglobin in the tumour tissue was replaced by deoxyhaemoglobin at that time. After 1 h, the reflectance spectrum of the tumour regained the characteristics seen in the pre-PDT treatment spectrum with the reappearance of 542 and 577 nm troughs. These changes in oxyhaemoglobin/deoxyhaemoglobin levels are more clearly presented by ratioing the post-PDT spectrum to pre-PDT spectrum (Figure 3B). The values around 542 and 577 nm are now depicted as prominent peaks emphasising the reduction in oxyhaemoglobin levels immediately after PDT compared to pre-PDT values. This drop in haemoglobin oxygen saturation obviously does not persist, since ratioing the reflectance spectrum taken at 1 h after PDT to the spectrum taken before PDT reveals no peaks around 542 and 577 nm; in fact, a small trough at 577 nm hints that oxygen levels might have even exceeded the pre-PDT values.

**Figure 4:**
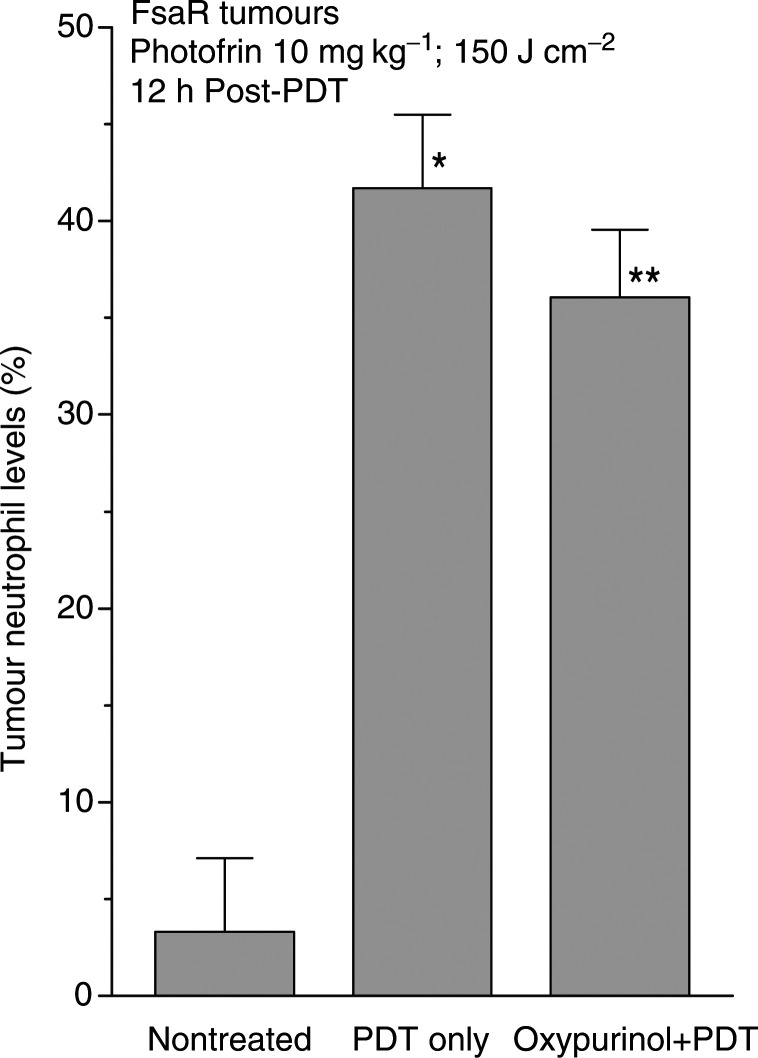
The effect of oxypurinol on the accumulation of neutrophils in PDT treated tumours. Subcutaneous FsaR tumours were PDT treated as described in Figure 1 and oxypurinol treatment was as described in Figure 2. Following their excision at 12 h post-PDT, the tumours were dissociated into cell suspensions, stained with antibodies for neutrophil identification, and analysed by flow cytometry. The results are presented as the percentage of neutrophils in total tumour cell populations. Bars are s.d., *n*=4; ^*^depicts statistically significant difference from the level in nontreated tumours (*P*<0.001), ^**^depicts statistically significant difference from the level in PDT-only group (*P*<0.05).

Proinflammatory effects associated with XO activity are known to stimulate local neutrophil sequestration (Zimmerman and Granger, 1994), and it is also well established that PDT induces neutrophil accumulation in the treated tumours (Krosl *et al*, 1995; Gollnick *et al*, 1997; Sun *et al*, 2002). The oxypurinol treatment that inhibits XO activity produced a decrease in the levels of neutrophils found in PDT-treated tumours (Figure 4

). Flow cytometry-based analysis of cell suspensions disaggregated from FsaR tumour tissue reveals that nontreated tumours contained only a minor neutrophil population (around 3%), which raised dramatically following PDT treatment. The time point of 12 h post-PDT depicted in Figure 4 is within the period of peak levels of the PDT-induced neutrophil invasion with the FsaR tumor model (Cecic, de Vit, Sluiter and Korbelik, unpublished results). While over 40% of cells in PDT-treated tumours at that time point were neutrophils, this level decreased significantly, although not dramatically, in the samples obtained from mice treated with oxypurinol.

**Figure 5:**
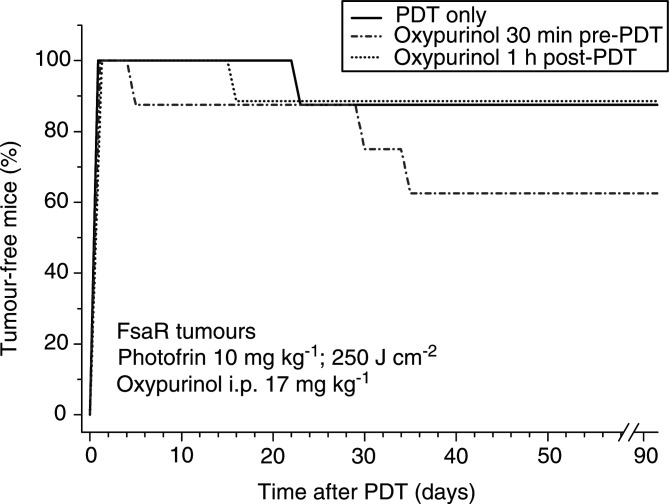
The effect of oxypurinol on the PDT response of FsaR tumours. Subcutaneous FsaR tumours were PDT treated as in Figure 2. Oxypurinol (17 mg kg^−1^ i.p.) was given to mice either 30 min before or 1 h after PDT light treatment. The mice were thereafter monitored for 90 days for signs of tumour growth. The difference in response between PDT-only group and PDT preceded by oxypurinol group is statistically significant (*P*<0.05).

The impact of oxypurinol treatment on PDT response of FsaR tumours is shown in Figure 5

. The chosen PDT dose resulted in a rapid ablation of treated tumours, and only a minor fraction of these lesions recurred several weeks later. The oxypurinol administration performed before PDT showed no significant effect on the initial PDT response, but it increased the rate of tumour recurrence and this resulted in statistically significant decline in tumour cures. In contrast, the administration of oxypurinol at 1 h after PDT exhibited no significant effect on the tumour response to PDT.

## DISCUSSION

Treatment of FsaR tumours with Photofrin-based PDT results in a burst of elevated XO activity peaking immediately after the termination of photodynamic light delivery that rapidly declines and fades away within 30 min post-PDT (Figure 1 and Figure 2). This phenomenon correlates with the blood reperfusion in PDT-treated tumours occurring after an ischaemic period induced during the light treatment (Korbelik *et al*, 2000; van Geel *et al*, 1994). A combination of several factors is probably responsible for the generation of ischaemia in PDT-targeted tissues. One is the depletion of oxygen through its consumption by the photodynamic process (Foster *et al*, 1991; Bush *et al*, 2000). Also contributing is the reduced blood flow consequent to the vasoconstriction caused by inflammatory mediators like thromboxane whose release is known to be induced by PDT (Fingar *et al*, 1990,^1992^), and blood flow stasis resulting from microvascular damage or obstruction by adhering neutrophils and platelets (Star *et al*, 1986; Henderson and Fingar, 1989; Fingar *et al*, 1992). As soon as its consumption drops with the termination of light delivery, oxygen will diffuse from the surrounding tissues into the ischaemic region. The reoxygenation will be supported by the vasodilating effect of mediators such as histamine and prostaglandins (Kamide *et al*, 1984; Henderson and Donovan, 1989; Henderson and Dougherty, 1992), and nitric oxide (NO) produced by activated neutrophils (Cecic and Korbelik, 2002).

The occurrence of the above-discussed changes in tumour oxygenation is supported by the evidence obtained using visible reflectance measurement with FsaR tumours (Figure 3). Diffuse reflectance spectroscopy probes the scattering and absorption properties of tissue. Haemoglobin, one of the main chromophores in well-perfused tissues in the visible range, is characterised by the strong absorption bands of its oxygenated form (oxyhaemoglobin) at 542 and 577 nm (Zonios *et al*, 2001). These bands are lost when oxyhaemoglobin is converted into deoxyhaemoglobin under reduced oxygen concentrations, which enables the development of visible reflectance technique for noninvasive *in vivo* monitoring of changes in tissue oxygenation. In the spectral range 500–600 nm, the tissue penetration depth with this technique is up to 1 mm (Hillenkamp, 1979). This makes it adequate for monitoring the oxygenation of subcutaneous lesions such as FsaR tumours, particularly in mouse models since the skin overlying the tumours (<200 *μ*m) is thinner than human skin. The reflectance spectra that we obtained with a series of FsaR tumours before and after PDT were remarkably consistent and highly reproducible. At this point, presented are qualitative characteristics of dramatic PDT-induced changes in tumour oxygenation. They demonstrate that the oxygenation of FsaR tumours is markedly reduced at the end of PDT light delivery, but is restored to pretreatment or even higher values 1 h later (when these tumours were showing signs of a strong oedema). We are currently developing a ratio technique for processing data from pre- and post-PDT treatment spectra (based on a modification of the model described by Zonios *et al*, 2001) for deriving quantitative values for changes in tumour oxygenation. We have also carried out a preliminary examination of oxygen tension in FsaR tumours with Eppendorf pO_2_ histograph. These measurements indicate that nontreated FsaR tumours are moderately oxygenated (average 39.5 mmHg) and that there is a greater than two-fold increase in these levels at 1 h post-PDT treatment as used in the reflectance spectrum protocol (M Korbelik, AI Minchinton, J Sun, unpublished results). Obviously, more investigation is warranted on the kinetics of oxygenation and blood perfusion changes in PDT-treated tumours as this is directly linked with the mechanism of antitumour effect of this modality, but such a task goes well beyond the scope of the present paper.

The reperfusion following an ischaemic episode is a well-recognised indication for the development of a classical physiological insult, the I/R injury (Zimmerman and Granger, 1994; Kilgore *et al*, 1999). This event is hallmarked by the burst in XO activity such as demonstrated in this report. Therefore, it can be concluded that the I/R injury was induced in PDT-treated FsaR tumours. The occurrence of this insult can obviously have important implications for the response of PDT-treated tumours.

Intense generation of superoxide mediated by XO is largely responsible for the damage inflicted by I/R injury (Parkins *et al*, 1998). The endothelium of the vasculature in PDT-treated tumours could sustain a heavy damage from the released superoxide and such event will have a powerful proinflammatory impact. The I/R injury is known to be associated with the activation of complement (a potent instigator of the inflammatory process whose engagement was recently demonstrated in PDT-treated tumours (Cecic and Korbelik, 2002)), proadhesive changes in the endothelium, release of various inflammatory mediators, PARP activation, and influx of activated neutrophils (Szabo and Dawson, 1998; Kilgore *et al*, 1999). Oxypurinol, which inhibits the PDT-induced XO activation (Figure 2), also attenuates the accumulation of neutrophils in PDT-treated tumours (Figure 4). In an earlier report, we have shown that the oxypurinol treatment reduces, as well, the extent of systemic neutrophilia that develops in mice bearing PDT-treated tumours (Cecic and Korbelik, 2002). These observations suggest that the impact from I/R injury contributes to the induction of neutrophil invasion into the tumours treated by PDT.

Evidence that superoxide formation is associated with PDT treatment was provided in several earlier studies. In addition to our finding that intravenous SOD administration immediately after photodynamic light treatment decreases the cure rate of PDT-treated mouse FsaR and EMT6 tumours (Korbelik *et al*, 2000), Athar *et al* (1989) have shown that the effect of PDT on mouse skin can be augmented by an SOD inhibitor and diminished by an SOD mimic. Using ESR spectroscopy, the superoxide production was also documented in PDT-treated tissues (Athar *et al*, 1988) and in photoirradiated aqueous photosensitiser liposomal preparations (Hajdur *et al*, 1997a,1997b).

Superoxide and its dismutation product hydrogen peroxide have been described as stimulators of transcriptional activation of stress proteins (Demple *et al*, 1999). On the other hand, PDT-generated tumour ischaemia can elicit a specific stress response known to activate hypoxia-inducible factor-1 (HIF-1) that can be responsible for the induction of VEGF expression (Ferrario *et al*, 2000), and activation of early response genes including cyclooxygenase-2 and inducible nitric oxide synthase (Hierholzer *et al*, 2001). Thus, in addition to the primary stress response triggered by singlet oxygen generated directly in photodynamic reactions (Dougherty *et al*, 1998), several forms of secondary stress may be inflicted in PDT-treated tumours: oxidative stress mediated by superoxide, oxidative and nitrosative stress mediated by NO (Korbelik and Cecic, 2003), and hypoxic stress. The extent of PDT-induced injury and its contribution to the antitumour effect of this modality is likely to differ depending on the type of treated tumour and photosensitiser class used for PDT. It may also be influenced by the duration of PDT light treatment and the dose rate, although we have not observed significant differences following the treatment with 150 and 250 J cm^−2^, both delivered at around 110 mW cm^−2^ (Figure 1 and Figure 2). The rate of xanthine dehydrogenase conversion into XO during ischaemia differs in various tissues. In the ileum, nearly complete conversion occurs within 10 s, whereas in the heart XO levels double after 8 min of nonperfusion (McCord, 1985). Similar differences can be expected to exist among different types of tumours. Therefore, the extent of PDT-induced I/R injury, which with FsaR tumours is obviously contributing to the therapy outcome (Figure 5), is likely to vary in different types of lesions. With respect to the photosensitisers used for PDT, in addition to the XO activation following Photofrin-based PDT described in this study, we have evidence of a similar effect produced by benzoporphyrin derivative-based PDT (Korbelik and Cecic, 2003).

An important element that can influence the extent of I/R injury in PDT-treated tumours is the extent of endogenous NO production, which varies among solid human and animal tumours (Parkins *et al*, 1995; Tozer and Everett, 1997). NO reacts rapidly with superoxide forming peroxynitrite anion (Blough and Zafiriou, 1985; McCall *et al*, 1989) and this can result in superoxide detoxification (Wink *et al*, 2001). This element may contribute to the observed tendency of an increased resistance to PDT of tumours characterised by elevated intrinsic NO production (Korbelik *et al*, 2000). Low NO-producing tumours were shown to be more profoundly affected by transient clamping of blood vessels feeding subcutaneous tumours that results in I/R injury associated with substantial tumour cytotoxicity (Parkins *et al*, 1985, 1988).

In conclusion, this work demonstrates that PDT can induce I/R injury and the extent of this insult may be sufficiently pronounced to have an important impact on the therapy outcome. The infliction of I/R injury is a classical proinflammatory event, and we suggest that it participates in the induction of inflammatory response that has a major role in the antitumour effect of PDT (Korbelik and Cecic, 2003).

## References

[bib1] Athar M, Elmets CA, Bickers DR, Mukhtar H (1989) A novel mechanism for the generation of superoxide anions in hematoporphyrin derivative-mediated cutaneous photosensitization. Activation of xanthine oxidase pathway. J Clin Invest 83: 1137–1143 2539390 10.1172/JCI113993 PMC303799

[bib2] Athar M, Mukhtar H, Elmets CA, Zaim MT, Lloyd JR, Bickers DR (1988) *In situ* evidence for the involvement of superoxide anions in cutaneous porphyrin photosensitization. Biochem Biophys Res Commun 151: 1054–1059 2833253 10.1016/s0006-291x(88)80472-8

[bib3] Blough NV, Zafiriou OC (1985) Reaction of superoxide with nitric oxide to form peroxynitrate in alkaline aqueous solution. Inorg Chem 24: 3504–3505

[bib4] Bush TM, Hahn SM, Evans SM, Koch CJ (2000) Depletion of tumor oxygenation during photodynamic therapy: detection by the hypoxia marker EF3 [2-(2-Nitroimidazol-1[*H*]-yl)-*N*-(3,3,3-trifluoropropyl)acetamide]. Cancer Res 60: 2636–2642 10825135

[bib5] Cecic I, Korbelik M (2002) Mediators of peripheral blood neutrophilia induced by photodynamic therapy of solid tumors. Cancer Lett 183: 43–51 12049813 10.1016/s0304-3835(02)00092-7

[bib6] Cecic I, Parkins CS, Korbelik M (2001) Induction of systemic neutrophil response in mice by photodynamic therapy of solid tumors. Photochem Photobiol 74: 712–720 11723800 10.1562/0031-8655(2001)074<0712:iosnri>2.0.co;2

[bib7] De Greef KE, Ysebaert DK, Ghielli M, Vercauteren S, Nouwen EJ, Eyskens EJ, De Broe ME (1998) Neutrophils and acute ischemia-reperfusion injury. J Nephrol 11: 110–122 9650119

[bib8] Demple B, Hidalgo E, Ding H (1999) Transcriptional regulation via redox-sensitive iron–sulphur centres in an oxidative stress response. Biochem Soc Symp 64: 119–128 10207625

[bib9] Dougherty TJ, Gomer CJ, Henderson BW, Jori G, Kessel D, Korbelik M, Moan J, Peng Q (1998) Photodynamic therapy. J Natl Cancer Inst 90: 889–905 9637138 10.1093/jnci/90.12.889 PMC4592754

[bib10] Ferrario A, von Thiel KF, Rucker N, Schwarz MA, Gill PS, Gomer CJ (2000) Antiangiogenic treatment enhances photodynamic therapy responsiveness in a mouse mammary carcinoma. Cancer Res 60: 4066–4069 10945611

[bib11] Fingar VH, Wieman TJ, Doak KW (1990) Role of thromboxane and prostacyclin release on photodynamic therapy-induced tumor destruction. Cancer Res 50: 2599–2603 2139357

[bib12] Fingar VH, Wieman TJ, Wiehle SA, Cerrito PB (1992) The role of microvascular damage in photodynamic therapy: the effect of treatment on vessel constriction, permeability, and leukocyte adhesion. Cancer Res 52: 4914–4921 1387584

[bib13] Foster TH, Murant RS, Bryant RG, Knox RS, Gibson SL, Hilf R (1991) Oxygen consumption and diffusion effects in photodynamic therapy. Radiat Res 126: 296–303 2034787 10.2307/3577919

[bib14] Gollnick SO, Liu X, Owczarczak B, Musser DA, Henderson BW (1997) Altered expression of interleukin 6 and interleukin 10 as a result of photodynamic therapy *in vivo*. Cancer Res 57: 3904–3909 9307269

[bib15] Granger DN, McCord JM, Parks DA, Hollwarth ME (1986) Xanthine oxidase inhibitors attenuate ischemia-induced vascular permeability changes in the cat intestine. Gastroenterology 90: 80–84 3753555 10.1016/0016-5085(86)90078-8

[bib16] Hajdur C, Wagnières G, Ihringer F, Monnier P, van Den Bergh H (1997a) Production of the free radicals O_2_·^−^ and ·OH by irradiation of the photosensitizer zinc(II) phthalocyanine. J Photochem Photobiol B: Biol 38: 196–202 10.1016/s1011-1344(96)07440-4 9203381

[bib17] Hajdur C, Wagnières G, Monnier P, van Den Bergh H (1997b) EPR and spectrophotometric studies of free radicals (O_2_·^−^, ·OH, BPD-MA·^−^) and singlet oxygen (^1^O_2_) generated by irradiation of benzoporphyrin derivative monoacid ring A. Photochem Photobiol 65: 818–827

[bib18] Henderson BW, Donovan JM (1989) Release of prostaglandin E_2_ from cells by photodynamic treatment *in vitro*. Cancer Res 59: 6896–6900 2531034

[bib19] Henderson BW, Dougherty TJ (1992) How does photodynamic therapy work? Photochem Photobiol 55: 145–157 1603846 10.1111/j.1751-1097.1992.tb04222.x

[bib20] Henderson BW, Fingar VH (1989) Oxygen limitation of direct tumor cell kill during photodynamic treatment of a murine tumor model. Photochem Photobiol 49: 299–304 2525260 10.1111/j.1751-1097.1989.tb04110.x

[bib21] Hernandez LA, Grisham MB, Twohig B, Arfors KE, Harlan JM, Granger DN (1987) Role of neutrophils in ischaemia-/reperfusion-induced microvascular injury. Am J Physiol 253: H699–H703 3631303 10.1152/ajpheart.1987.253.3.H699

[bib22] Hierholzer C, Harbrecht BG, Billiar TR, Tweardy DJ (2001) Hypoxia-inducible factor-1 activation and cyclo-oxygenase-2 induction are early reperfusion-independent inflammatory events in hemorrhagic shock. Arch Orthop Trauma Surg 121: 219–222 11317684 10.1007/s004020000211

[bib23] Hillenkamp F (1979) Interaction between laser radiation and biological systems. In Lasers in Biology and Medicine, Hillenkamp F, Pratesi R, Sacci C (eds) pp 57–61. New York: Plenum Press.

[bib24] Kamide R, Gigli I, Lim HW (1984) Participation of mast cells and complement in the immediate phase of hematoporphyrin-induced phototoxicity. J Invest Dermatol 82: 485–490 6150958 10.1111/1523-1747.ep12261010

[bib25] Kilgore KS, Todd III RF, Lucchesi BR (1999) Reperfusion injury. In Inflammation: Basic Principles and Clinical Correlations, Galin JI, Snyderman R (eds) pp 1047–1060. Philadelphia: Lippincott Williams & Wilkins.

[bib26] Korbelik M, Cecic I (2003) Mechanism of tumor destruction by photodynamic therapy. In Handbook of Photochemistry and Photobiology, Nalwa HS (ed) Vol. 4, Chapter R. Stevenson Ranch, CA: American Scientific Publishers (in press)

[bib27] Korbelik M, Parkins CS, Shibuya H, Cecic I, Stratford MRL, Chaplin DJ (2000) Nitric oxide production by tumour tissue: impact on the response to photodynamic therapy. Br J Cancer 82: 1835–1843 10839299 10.1054/bjoc.2000.1157 PMC2363231

[bib28] Krosl G, Korbelik M, Dougherty GJ (1995) Induction of immune cell infiltration into murine SCCVII tumour by Photofrin-based photodynamic therapy. Br J Cancer 71: 549–555 7880738 10.1038/bjc.1995.108 PMC2033617

[bib29] Lentsch AB, Ward PA (2000) The NF*κ*B/I*κ*B system in acute inflammation. Arch Immunol Ther Exp (Warsz) 48: 59–63 10807044

[bib30] McCall TB, Boughton-Smith NK, Palmer RM, Whittle BJ, Moncada S (1989) Synthesis of nitric oxide from L-arginine by neutrophils. Release and interaction with superoxide anion. Biochem J 261: 293–296 2549965 10.1042/bj2610293 PMC1138817

[bib31] McCord JM (1985) Oxygen derived free radicals in postischemic tissue injury. N Engl J Med 321: 159–163 10.1056/NEJM198501173120305 2981404

[bib32] Parkins CS, Dennis MF, Stratford MRL, Hill SA, Chaplin DJ (1995) Ischemia reperfusion injury in tumors: the role of oxygen radicals and nitric oxide. Cancer Res 55: 6026–6029 8521386

[bib33] Parkins CS, Holder AL, Dennis MF, Stratford MR, Chaplin DJ (1998) Involvement of oxygen free radicals in ischaemia-reperfusion injury to murine tumours: role of nitric oxide. Free Radic Res 28: 271–281 9688213 10.3109/10715769809069279

[bib34] Parks DA, Williams TK, Beckman JC (1988) Conversion of xanthine dehydrogenase to oxidase in ischemic intestine: reevaluation. Am J Physiol 254: G768–G774 3163236 10.1152/ajpgi.1988.254.5.G768

[bib35] Ryter SW, Gomer CJ (1993) NF*κ*B binding activity in mouse L1210 cells following Photofrin II mediated photosensitization. Photochem Photobiol 58: 753–756 8284329 10.1111/j.1751-1097.1993.tb04964.x

[bib36] Spector T, Hall WW, Krenitsky TA (1986) Human and bovine xanthine oxidases. Inhibition studies with oxypurinol. Biochem Pharmacol 35: 3109–3114 3755906 10.1016/0006-2952(86)90394-1

[bib37] Star WM, Marijnissen HPA, Berg-Blok AE, Versteeg JAC, Franken KAP, Reinhold HS (1986) Destruction of rat mammary tumor and normal tissue microcirculation by hematoporphyrin derivative photoradiation observed *in vivo* in sandwich observation chambers. Cancer Res 46: 2532–2540 3697992

[bib38] Sun J, Cecic I, Parkins CS, Korbelik M (2002) Neutrophils as inflammatory and immune effectors in photodynamic therapy-treated mouse SCCVII tumours. Photochem Photobiol Sci 1: 690–695 12665307 10.1039/b204254a

[bib39] Szabo C, Dawson VL (1998) Role of poly(ADP-ribose) synthetase in inflammation and ischemia-reperfusion. Trends Pharmacol Sci 19: 287–298 9703762 10.1016/s0165-6147(98)01193-6

[bib40] Tozer GM, Everett SA (1997) Nitric oxide in tumour biology and cancer therapy. Part 1: physiological aspects. Clin Oncol 9: 282–293 10.1016/s0936-6555(05)80061-5 9368723

[bib41] Van Geel IPJ, Oppelaar H, Oussoren YG, Stewart FA (1994) Changes in perfusion of mouse tumours after photodynamic therapy. Int J Cancer 56: 224–228 8314306 10.1002/ijc.2910560214

[bib42] Volpe JP, Hunter N, Basic I, Milas L (1985) Metastatic properties of murine sarcomas and carcinomas. I. Positive correlation with lung colonization and lack of correlation with s.c. tumor take. Clin Exp Metast 3: 281–294 10.1007/BF01585082 4075613

[bib43] Wink DA, Mirand KM, Espey MG, Pluta RM, Hewett SJ, Colton C, Vitek M, Feelisch M, Grisham MB (2001) Mechanisms of the antioxidant effects of nitric oxide. Antioxid Redox Signal 3: 203–213 11396476 10.1089/152308601300185179

[bib44] Workman P, Twentyman P, Balkwill F, Balmain A, Chaplin D, Double J, Embleton J, Newell D, Raymond R, Stables J, Stephens T, Wallace J (1998) United Kingdom Co-ordinating Committee on Cancer Research (UKCCCR) Guidelines for the Welfare of Animals in Experimental Neoplasia (2nd edn.). Br J Cancer 77: 1–10 10.1038/bjc.1998.1 PMC2151254 9459138

[bib45] Zeng H, MacAulay C, McLean DI, Lui H, Palcic B (1995) Miniature spectrometer and multi-spectral imager as a potential diagnostic aid for dermatology. Proc SPIE 2387: 57–61

[bib46] Zimmerman BJ, Granger DN (1994) Mechanisms of reperfusion injury. Am J Med Sci 307: 284–292 8160724 10.1097/00000441-199404000-00009

[bib47] Zonios G, Bykowski J, Kollias N (2001) Skin melanin, hemoglobin, and light scattering properties can be quantitatively assessed *in vivo* using diffuse reflectance spectroscopy. J Invest Dermatol 117: 1452–1457 11886508 10.1046/j.0022-202x.2001.01577.x

